# Digital support for caregivers of patients with non-communicable diseases during COVID-19: Lessons from a cancer case study in Vietnam

**DOI:** 10.7189/jogh.11.03095

**Published:** 2021-08-07

**Authors:** Hien Thi Ho, Chris Jenkins, Hung Quang Ta, Chi Linh Bui, Minh Van Hoang, Olinda Santin

**Affiliations:** 1 Faculty of Clinical Medicine, Hanoi University of Public Health, Hanoi, Vietnam; 2 Centre for Public Health, Institute of Clinical Sciences, Queen’s University Belfast, Belfast, Northern Ireland, UK; 3 Department of Mathematics and Computer Science, Hanoi University, Hanoi, Vietnam; 4 Hanoi University of Public Health, Hanoi, Vietnam; 5 School of Nursing and Midwifery, Queen’s University Belfast, Medical Biology Centre, Belfast, Northern Ireland, UK

Non-communicable diseases (NCDs) cause more than 71% of global deaths, of which 77% were reported from low- and middle- income countries (LMICs) [1]. In LMICs, such as Vietnam, this population is largely dependent on informal caregivers (usually family caregivers) who provide essential care without information or sufficient support [2] and do so whilst managing complex needs. As the number of COVID-19 cases rapidly increases in Vietnam and with social restrictions, many caregivers are forced to provide complex care and support at home with limited health literacy and little contact with the health service. COVID-19 has highlighted the urgent requirement for digitalized health care, however, this movement is in its infancy in Vietnam and is hindered by a series of challenges [3].

As Vietnam moves rapidly to provide care and support for caregivers affected by NCDs during COVID-19, our international team wishes to offer our key reflections and experience of understanding the needs of the families affected by cancer and developing feasible digital solutions.

## REFLECTIONS

### 1. The growing problems of NCDs cannot be addressed by the current health system – we urgently need to continue to work together

In Vietnam, the rising burden of NCDs (ie, cancers) is a major health concern, accounting for 77% of deaths in the country [1]. In particular, Vietnam has observed a rapid increase in cancer burden with 182 563 new cases and 122 690 deaths in 2020 [4]. Despite this rise, the health service is currently not fit for purpose. The current health service lacks resources, personel, equipment and in some cases quality of care. Many people have limited access to primary care, with those in public hospitals being treated in overcrowded environments. As COVID-19 cases rise and the potential risks of transmission increase for those with NCDs, there is a concern that many families will be forced to manage at home with no access to primary care or hospital.

As an international research community, COVID-19 has intensified the need for us to work together to improve the services for those with NCDs in LMICs. We need to increase international research and health service dialogue so that we can learn and work with each other to provide meaningful solutions.

### 2. Caregivers are “invisible” but are key in recovery for patients with NCDs: their unmet needs should be addressed

As the prevalence of NCDs rises, so do the number of people providing informal care. Informal caregivers are of particular importance to the delivery of care for Vietnamese NCDs patients. First, caregivers undertake multiple caring roles for patients, helping access health care, making treatment decisions, providing informational and emotional support, and basic care needs (ie, daily care, nutrition) [2,5]. Second, due to the inter-dependence of patient and caregivers’ health, supporting caregivers has the potential to impact upon patients’ health and well-being.

Providing long-term care can have a profoundly negative impact on caregivers’ health, particularly as the patients’ health declines. Their physical and psychological health, however, are not the priority for themselves or the current health system.

This large population of informal caregivers is currently without sufficient information and support. Our recent study showed the high level of unmet needs of caregivers of cancer patients in oncology hospitals of Vietnam. This is mainly due to the overcrowding of central hospitals and the central focus on the patients’ medical treatment needs [6]. Furthermore, as hospital infrastructure struggles to meet the demands of diagnosis and treatments especially in COVID-19, the majority of the care responsibility (including inpatient care) falls on the caregivers. Palliative and supportive care services, social work, counselling, and support and information services, all of which are integral to holistic cancer services in high-income countries, are underdeveloped.

Prior to the outbreak, the burden of cancer on informal caregivers was already demonstrated to be significant [2]. In the context of COVID-19, this burden will increase. Although some hospitals and treatments have had periods of closing/re-commencing some services, the pressure of a backlog, ongoing lockdowns, lack of access to support and vaccination rollout increases the need to rapidly develop an understanding of the impact of COVID-19 on caregivers and how they can be supported remotely.

### 3. A increasing problem: new and additional challenges to cancer services posed by COVID-19 in Vietnam

Vietnam has successfully minimized the impact of COVID in terms of infections and deaths in the first stage, this has come at the cost of strict social distancing measures and restrictions, including on some hospitals. The current situation, however, is getting worse. As of June 22 of 2021, there was 13 530 cases with 69 deaths. One month later, the number of cases is 78 269 with 370 deaths [7].

**Figure:**
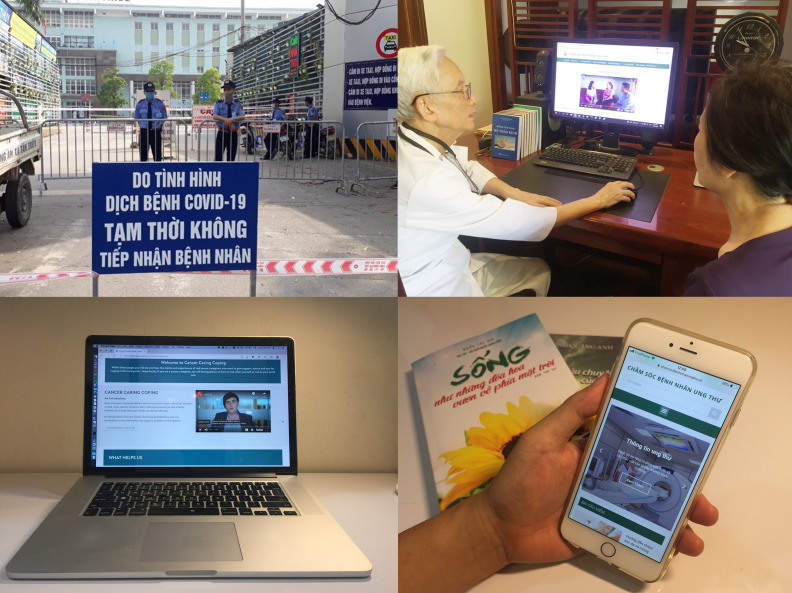
Photo: National Cancer Hospital under lockdown during COVID-19 pandemic and digital support for cancer caregivers (Source: https://laodong.vn/ and photos from studies conducted by the authors on cancer caregivers in UK and Vietnam).

In this context in which health services are disrupted, there are more challenges for caregivers and patients. These challenges include inability to travel to or access appointments, delays in treatments, social distancing restrictions in hospitals, affecting family members who can provide support, changing of services and worries regarding transmission risk due to immunosuppression.

Cancer services in Vietnam are significantly affected. Oncology facilities have been locked down due to on-site outbreaks, examination and treatment of outpatients reduced, and for patients who have finished treatment, follow-up appointments have been restricted. Cancer patients experience poor physical and mental health. Cancer caregivers in Vietnam are usually responsible for multiple caring tasks, and in the context in which hospital services are limited, the role and importance of caregivers is particularly significant.

### 4. Digital resources don’t have all the answers but they could help!

The physical lockdown and the new waves of COVID-19 have demonstrated the need for support and care for patients and caregivers, particularly flexible, high quality health services that can be delivered remotely. Therefore, digital resource development could be a feasible, effective approach to provide interventions for all NCD caregivers in both Vietnam and similar contexts globally. Online delivery has high potential for reach, with 69.2 million internet users (72.9% of the population) in Vietnam [8]. An online resource may provide access to support and information to large numbers of people across several locations in a cost-effective way.

Our recent study on cancer caregivers in Vietnam showed that the development of a web-based resource was identified as an urgent need. A web-based resource was viewed as a suitable interface to provide support across regions in a sustainable way [5]. The study demonstrates that for cancer, caregivers and health care professionals are supportive of a web-based intervention whilst recognising that this mode of delivery has limitations particularly in terms of accessibility for older caregivers and those that live in very remote locations.

## LESSONS FROM VIETNAM: HOW TO DEVELOP WEB-BASED RESOURCES

To date, support for interventions for caregivers that utilise online technology have relied on a methodologically weak evidence base [9]. Recent research, however, has indicated the potential for these types of interventions. Our approach for interventions has been used to develop digital resources for caregivers for different groups of NCDs patients in Vietnam.

We have found that intervention development is best served through co-development with multiple stakeholders to draw on their experiences and identify improvements to services. We have experienced a high level of motivation across multiple regions of Vietnam for patient- and caregiver- lead service/support development. We have developed a prototype of a digital support Vietnam-Cancer Caring Coping (V-CCC) using a co-design method for caregivers of cancer patients. This intervention follows the web-based CCC in UK in which an online resource was co-designed by caregivers for cancer caregivers in response to their unmet needs [10]. There is increasing evidence for the use of online interventions to support cancer caregivers, particularly due to their wide reaching and sustainable mode of delivery [9].

### Proposed framework for web-based interventions

We have developed a two-stage framework using co-design approach with modifications which may be considered for the rapid development of user-led digital resources. It should be noted that modifications are needed to tailor the needs of resources in different settings.

1. Phase 1: Development of the web resources
  1. Step 1: Organize workshops with caregivers and quarterly meetings with an expert advisory team.
  2. Step 2: Development of web prototype.
  3. Step 3: User testing: an unstructured feedback session with caregivers to obtain their information on the resource.
  4. Step 4: Refining prototype: changes of the resource component were made to the resource based on the feedback.
2. Phase 2: Web resource user testing
  1. Step 5: Evaluation of the resource using multiple-method design was conducted. A pre- and post- mixed method evaluation of the digital resources on patients and caregivers’ experiences were conducted.
  2. Step 6: Development of the final resource.

## CONCLUSIONS AND RECOMMENDATIONS

This viewpoint paper highlights the urgent needs for support for caregivers of NCD patients in Vietnam and offers a novel approach for developing a co-designed digital resource for them. The digital approach is particularly relevant during COVID-19 when both health services and support to patients and caregivers have been disrupted. This approach is transferable and contextually adaptable, and offers the potential to inform and support caregivers in many other LMICs.
